# Antidepressant and anxiolytic activity of *Lavandula officinalis* aerial parts hydroalcoholic extract in scopolamine-treated rats

**DOI:** 10.1080/13880209.2017.1285320

**Published:** 2017-02-06

**Authors:** Batool Rahmati, Zahra Kiasalari, Mehrdad Roghani, Mohsen Khalili, Fariba Ansari

**Affiliations:** a Neurophysiology Research Center, Shahed University, Tehran, Iran;; b Department of Physiology, School of Medicine, Shahed University, Tehran, Iran

**Keywords:** Memory impairment, Y maze, elevated plus maze, forced swimming, depression, anxiety

## Abstract

**Context:** Anxiety and depression are common in Alzheimer’s disease (AD). Despite some evidence, it is difficult to confirm *Lavandula officinalis* Chaix ex Vill (Lamiaceae) as an anxiolytic and antidepressant drug.

**Objective:** The effects of *L. officinalis* extract were studied in scopolamine-induced memory impairment, anxiety and depression-like behaviour.

**Materials and methods:** Male NMRI rats were divided into control, scopolamine alone-treated group received scopolamine (0.1 mg/kg) intraperitoneally (i.p.), daily and 30 min prior to performing behavioural testing on test day, for 12 continuous days and extract pretreated groups received aerial parts hydro alcoholic extract (i.p.) (100, 200 and 400 mg/kg), 30 min before each scopolamine injection. Memory impairment was assessed by Y-maze task, while, elevated plus maze and forced swimming test were used to measure anxiolytic and antidepressive-like activity.

**Results:** Spontaneous alternation percentage in Y maze is reduced by scopolamine (36.42 ± 2.60) (*p* ≤ 0.001), whereas lavender (200 and 400 mg/kg) enhanced it (83.12 ± 5.20 and 95 ± 11.08, respectively) (*p* ≤ 0.05). Also, lavender pretreatment in 200 and 400 mg/kg enhanced time spent on the open arms (15.4 ± 3.37 and 32.1 ± 3.46, respectively) (*p* ≤ 0.001). On the contrary, while immobility time was enhanced by scopolamine (296 ± 4.70), 100, 200 and 400 mg/kg lavender reduced it (193.88 ± 22.42, 73.3 ± 8.25 and 35.2 ± 4.22, respectively) in a dose-dependent manner (*p* ≤ 0.001).

**Discussion and conclusion:** Lavender extracts improved scopolamine-induced memory impairment and also reduced anxiety and depression-like behaviour in a dose-dependent manner.

## Introduction

Depression is one of the top five most prevalent diseases worldwide. By 2020, it is expected to be the second-leading cause of disability globally. Depression is typically presented as lowered mood, difficulty in thinking, loss of interest and physical complaints such as headache, disturbed sleep, loss of energy and change in sex drive (Dwyer et al. 2011; Tegegne et al. 2015). Previous studies have shown that as many as 80–90% of individuals with major depressive disorder (MDD) report symptoms of anxiety (Fißler & Quante 2014). Comorbid anxiety with depression predicts poor outcomes with a higher percentage of treatment resistance than either disorder occurring alone. Overlap of anxiety and depression complicates diagnosis and renders treatment challenging (Coplan et al. 2015). Also, it is reported that anxiety and depression are common in Alzheimer’s disease (AD), and is related with the duration, greater severity of dementia and lower education levels (Garca-Alberca et al. 2011). Anxiety disorders are a prevalent mental health problem in older age with a considerable impact on quality of life. Longitudinal studies indicate that elderly suffering from anxiety disorders have a high risk of relapse and persistence alongside the progression to depression and mixed anxiety depression states (Kasper 2015).

While there are many potential precipitating factors, it is currently believed that depression is primarily the result of biochemical alterations in the brain. Pharmaceutical treatments, including selective serotonin reuptake inhibitors (SSRI), tricyclic antidepressants (TCA) and monoamine oxidase inhibitors (MAOI), cause alterations in brain chemistry through neurotransmitter amplification and regulation and have been shown to be effective in the treatment of depression (Dwyer et al. 2011). Also, drug treatment of anxiety is predominantly carried out using antidepressant drugs (Kasper 2015). However, a number of adverse reactions occur with pharmaceutical antidepressant administration, including anticholinergic effects, gastrointestinal effects including nausea and constipation, orthostatic hypotension, arrhythmias, weight gain and sexual dysfunction (Dwyer et al. 2011). Thus, there is a need to search for new compounds and strategies of treatment that could improve conventional therapies. In this context, natural product research has been considered as an option for the development of drugs with innovative mechanisms of action and/or conceivably minimized adverse side effects (Müller et al. 2012). The use of plants and plant extracts to treat diseases is a therapeutic modality. According to the World Health Organization (WHO), about three-quarters of the world population relies upon traditional remedies (mainly herbs) for the health care of its people (Gilani & Rahman 2005).

*Lavandula officinalis* Chaix ex Vill (Lamiaceae) (syn. *Lavandula angustifolia* Mill.) tincture, commonly known as English Lavender or Ustukhuddoos, has long been used in Iranian traditional medicine for some nervous disorders such as epilepsy and depression (Ebn-e 1991). Based on the experimental and clinical studies, *L. officinalis* generally has been considered as a sedative, antidepressive, antispasmodic, antiflatulent, antiemetic, diuretic, anticonvulsant, antibacterial, analgesic and a general tonic (LaGow et al. 2004). Clinical trials have shown that patients with symptoms of anxiety and insomnia benefit from lavender oil aromatherapy and massages (Fißler & Quante 2014). Also, clinical trials have explored the antidepressant effects of lavender oil, showing that lavender oil used in aromatherapy has a positive effect on mood and can intensify relaxation as observed in the increased β power in EEG activity (Fißler & Quante 2014). It is reported that lavender oil has a significant effect on anxiety-related symptoms by significantly reducing restlessness and agitation in patients with sub-syndromal generalized anxiety disorder (Fißler & Quante 2014). Clinical trial also determined that a combination of imipramine and *L. angustifolia* was more effective than imipramine alone (Akhondzadeh et al. 2003). It is reported that exposure to lavender odour may have an anxiolytic profile in gerbils similar to the anxiolytic effects of diazepam. In addition, prolonged, 2-week lavender odour exposure increased exploratory behaviour in females indicating a further decrease in anxiety in this sex (Bradley et al. 2007). Lavender cream with foot-bath or alone can be used for pregnant women for reducing their stress, anxiety and depression (Effati-Daryani et al. 2015).

Serotonin neurotransmission, likely through 5-HT_1A_ receptors, participates in the pharmacological mechanism by which lavender essential oil exerts its anxiolytic-like effect (Chioca et al. 2013). Despite some evidence of anxiolytic and antidepressant effects of *L. officinalis*, it is difficult to confirm lavender as an anxiolytic drug. Methodological issues limit the extent to which any conclusions can be drawn regarding the efficacy/effectiveness of lavender. The best evidence suggests that oral lavender supplements may have some therapeutic effects. However, further studies are needed before firm conclusions can be drawn (Perry et al. 2012). While in humans, cognitive mechanisms of odour transduction (aromatherapy) may complicate the study of pharmacological effects, animal studies give some evidence for herbal medicine extract having pharmacological properties.

Scopolamine is a non-selective muscarinic receptor antagonist and it consistently has produced emotional-based learning impairment in rodents (Popović et al. 2015). Scopolamine has been used as a standard**-**reference drug for inducing age- and dementia-related cognitive deficits in healthy humans and animals. The pharmacological model of cholinergic amnesia using scopolamine became popular after the cholinergic hypothesis was formed (Klinkenberg & Blokland 2010).

Anxiety and depression are common in dementia including in Alzheimer’s disease. It is demonstrated that inhalation of dried flower heads lavender essential oil vapours improved scopolamine-induced spatial memory impairment and exhibited anxiolytic and antidepressant-like effects in scopolamine-treated rats (Hritcu et al. 2012). It is reported that the main constituents of lavender oil are linalool, linalyl acetate, cineole, terpinen-4-ol and camphor (Hritcu et al. 2012). These constituents can vary significantly in different parts of plant such as flower, leaves, stem and branches. The pure oil is most often used in aromatherapy and massage. Despite its popularity and long tradition of use, only recently scientifically based investigations into the biological activity of the various *Lavandula* products (essential oils or kind of extracts) have been undertaken to a greater extent. The biological actions of many of the chemical compounds found in lavender are not well understood (Hritcu et al. 2012). Therefore, the present study for the first time investigates the effect of *L. officinalis* aerial parts hydro-alcoholic extract on anxiety- and depression-like behaviour in scopolamine-treated rats.

## Materials and methods

### Animals

Fifty male NMRI rats weighing 250 ± 50 g at the start of the experiment were used. Animals were purchased from Laboratory Animals Sciences Center of Medical Sciences Baqiyatallah (Iran, Tehran). The animals were housed in plastic cages (26.5 × 42 × 15 cm), five rats per cage and kept under a 12 h light/dark cycle with lights on at 6 a.m. at a constant temperature of 21° ± 2 °C with free access to standard certified rodent diet and tap water and humidity 30–40%. In order to animal adaptation to a new environment, before any trial, animals were kept in animal house in medical faculty of Shahed University for a week. The experimental protocol was approved by Ethics Committee of Shahed University.

### Category animals

The rats were divided into five groups (10 animals per group): (1) control group received saline treatment (0.9% NaCl); (2) Scopolamine (Sco) alone-treated group received scopolamine (Sigma, Chicago, IL) 30 min after saline pretreatment daily, for 12 continuous days and 30 min prior to performing behavioural testing on test day (Fernandes et al. 2008; Hritcu et al. 2012; Xiang et al. 2012) at a dose of 0.1 mg/kg intraperitoneally (Kay et al. 2010); (3), (4), and (5) *L. officinalis* extract-pretreated groups received *L. officinalis* extract with concentrations of 100, 200 and 400 mg/kg (Kashani et al. 2011; Rahmati et al. 2012, 2013), per day, 30 min before each scopolamine injection and 1 h before each behavioural testing on test day. Materials were administered intraperitoneally (i.p.) in a volume of 0.3 mL. Scopolamine (0.1 mg/kg) was used for the induction of memory impairment, anxiety and depression-like behaviour for 12 continuous days (Hritcu et al. 2012).

### Drugs

Scopolamine hydro bromide (Sigma, Chicago, IL) was dissolved in sterile isotonic saline (0.9% NaCl) and administered i.p.

Dried aerial parts of *L. officinalis* (500 g) were purchased from the local market and were identified by Professor Amin, the Head of Herbarium of Faculty of Pharmacy, Tehran Medical Sciences of University, where a voucher specimen number was deposited under the reference no. PMP-314. *Lavandula officinalis* powder was prepared using a grinder. Then, plant powder was soaked in double-distilled water and ethanol 70% (1:4) for 3 d in a dark place at room temperature (25 °C) and filtered. Filtration was repeated three times. All filtrates evaporated to dryness in water bath at 60 °C. The yield of the extract was about 12%. It was stored at −20 °C until test day. Extract was dissolved and diluted in normal saline on the day of experiment.

### Behavioural testing apparatus

#### Y-maze task

Scopolamine-induced memory impairment was assessed by Y-maze task on the ninth day. Short-term memory was studied by spontaneous alternation behaviour in the Y-maze task. The Y maze used in the present study consisted of three arms (35 cm long, 25 cm high and 10 cm wide) and an equilateral triangular central area. Sixty minutes after lavender extract administration and 30 min after administration of scopolamine, rats were placed at the end of one arm and allowed to move freely through the maze for 8 min. The time limit in Y-maze test was 8 min, and every session was stopped after 8 min. An arm entry was counted when the hind paws of the rat were completely within the arm. Spontaneous alternation behaviour was defined as entry into all three arms on consecutive choices. The number of maximum spontaneous alternation behaviours was then the total number of arms entered minus 2 and percent spontaneous alternation was calculated as (actual alternations**/**maximum alternations) × 100 (Hritcu et al. 2012). Spontaneous alternation behaviour is considered to reflect spatial working memory, which is a form of short-term memory.

### Elevated plus-maze (EPM) task

On the 10th day, behaviour in the EPM is also utilized to measure exploration, anxiety and motor behaviour. The EPM consists of four arms, 49 cm long and 10 cm wide, elevated 50 cm above the ground. Two arms were enclosed by walls 30 cm high and the other two arms have no walls. On the 10th day of experiments, 30 min after the last drug administration, each rat was placed at the juncture of the open and closed arms and the amount of time spent on the open arms was recorded during a 5 min test. After each assay, the maze was carefully cleaned with wet tissue. Time spent on the open arms is an index of anxiolytic effects of drugs (Hritcu et al. 2012).

### Forced swimming test (FST)

The possible antidepressant effects of lavender extract were studied by the FST. On the first day of this test (adaptation day), animals were individually placed into non-transparent plastic cylindrical containers (diameter 30 cm, height 60 cm) containing 25 cm of water at a temperature of 25–26 °C. Rats were left to swim for 15 min before being removed, dried and returned to their cages. Testing was repeated after 24 h for 6 min (test day). This procedure was performed on the 11th and the 12th day 30 min after administration of the last drug. Test parameters on the test day were immobility times (time spent floating with the minimal movements to keep the head above the water) (Hritcu et al. 2012).

### Chimney test

Motor impairment was quantified with the ‘chimney test’. Motor coordination was also evaluated using the ‘chimney test’. In this test, rats had to climb backwards up a plastic tube (an inner diameter of 57 mm, a length of 452 mm), and motor impairment was indicated by the inability of the animals to climb backwards up the transparent tube within 60 s (Burda et al. 2011).

### Lavender chemical analysis

Essential oil was obtained by hydro-distillation of dried aerial parts of plant. Then, the chemical composition of essential oil was determined by gas chromatography–mass spectrometry (GC–MS) method. On this basis, its main ingredients were l-camphor (66%), eucalyptol or 1,8-cineol (4.4%), borneol (2.9%), fenchone (1.03%), α-linalool (0.93%), *cis*-α-terpineol (0.23%), myrtenal (1.24%), bornyl acetate (0.10%), caryophyllene oxide (1.66%), α-eudesmol (2.57%), 1*R*-α-pinene (0.50%) and camphene (0.69%).

However, the main active ingredients of lavender extract are seems to be non-volatile constituents, because a lot of volatile constituents of lavender, escape during the preparation of extract. Therefore, extract analysis also was performed.

### Extract analysis

#### Phytochemical screening tests

##### Preliminary phytochemical screening

Screening tests for flavonoids, alkaloids, tannins, saponins, cardiac glycosides and sterols were done based on the standard methods as follows:

##### Test for flavonoids

Flavonoids of the extracts are often detected by Cyanidin test. One gram of extract was dissolved in methanol (50%), HCl (37%) powder of magnesium and amyl alcohol (50%). Flavonoids appeared as orange to red zone. Extract of the Chamomile flowers was used as a standard of flavonoids (positive control) (Sharifzadeh et al. 2006).

##### Test for alkaloid

For the detection of alkaloids, three drops of Dragendroff reagents were added to the fraction that is separated by using HCl, NaCl, methanol and chloroform from 1 g of extract. Alkaloids appeared as brown, blue or whitish zone. A thin-layer chromatogram (TLC) spot test with Dragendroff, Wagner and Mayer’s reagents was performed for checking the results (Sharifzadeh et al. 2006).

##### Test for tannin

Plant extract (about 1.0 g) was stirred with sterile-distilled water (10 ml) and filtered (using Whatman number 1 filter paper). A blue colouration resulting from the addition of two drops of 10% FeCl_3_ reagent to the filtrate indicated the presence of tannins (pseudo tannins) (Abioye et al. 2013).

Also, according to the standard protocol, tannins were detected by adding NaCl (10%) and gelatin (1%) to the dissolved fraction of 1 g of extract in 20 ml of boiled water. The amount of precipitation showed the presence of tannins. As a standard positive control, the extract of *Quercus infectoria* fruits was used for tannins evaluation (Sharifzadeh et al. 2006).

##### Test for saponin

For saponins determination, the height of the foam produced after shaking the extract (1 g) in distilled water (10 ml) is one of the standard ways to determine the amount of saponins. Liquiritiae radix root extract was used as a standard for saponins (Sharifzadeh et al. 2006).

##### Test for cardiac glycosides

The extract (about 0.5 g) was dissolved in glacial acetic acid (2 mL) containing 1 drop of 1% FeCl_3_. This was underlaid with concentrated H_2_SO_4_. A brown ring at the interface indicated the presence of a deoxy sugar, a characteristic of cardiac glycosides. A violet ring may form just above the brown ring and gradually spreads through this layer (Abioye et al. 2013).

##### Test for sterols

Both Salkowski test and Liebermann–Burchard test were performed.

##### Salkowski test

2 ml of chloroform and 2 ml of concentrated H_2_SO_4_ was added to 2 ml of plant extract, and shaken well. The chloroform layer appeared red and the acid layer greenish yellow fluorescent. This confirms the presence of sterols.

##### Liebermann–Burchard test

2 ml of methanolic plant extract was mixed with chloroform. About 1–2 ml acetic anhydride and two drops of concentrated H_2_SO_4_ from the side of the test tube was added in the mixture. First red, then blue and finally green colour indicates the presence of sterols (Rimjhim et al. 2014).

Preliminary Phytochemical screening showed that Sterols, Flavonoids, pseudo tannins (FeCl_3_ test), Saponins and Cardiac glycosides are present in the extract, while Tannins and Alkaloids are absent.

##### Determination of total phenolic content

Total phenols were determined using Folin–Ciocalteu reagent as described by (Velioglu et al. 1998) with slight modifications. The extract (200 μL) was mixed with 1.5 ml of Folin–Ciocalteu reagent (previously diluted 10 times with distilled water) and allowed to stand at room temperature for 5 min. 1.5 ml sodium bicarbonate solution (60 g/L) was added to the mixture and after incubation for 90 min at room temperature, the absorbance level was measured at 750 nm using a UV–Visible spectrophotometer. Total phenolics were quantified by calibration curve obtained from measuring the absorbance of the known concentrations of Gallic acid standard solutions (25–150 μg/mL in 80% methanol). The results were calculated as Gallic acid equivalent (GAE) per 250 μg dry extract (Velioglu et al. 1998; Jia et al. 1999; Shams Ardekani et al. 2011).

##### Determination of total flavonoids content

Total flavonoids content was measured by the aluminium chloride colorimetric assay. An aliquot (1 mL) of extracts or standard solution of catechin (50, 100, 150, 200, 250 and 300 mg/L) was added to 10 ml volumetric flask containing 4 mL of double-distilled water. Then 0.3 mL 5% NaNO_2_ was added to the flask and, after 5 min, 0.3 ml AlCl_3_ (10%) was also added. At the sixth minute, 2 ml NaOH (1 M) was added and the total volume was made up to 10 ml with double-distilled water. The solution was mixed completely and the absorbance level was measured versus prepared reagent blank at 510 nm. Total flavonoids’s content was expressed as catechin equivalent (CE) per 500 μg dry extract (Table 3) (Velioglu et al. 1998; Jia et al. 1999; Shams Ardekani et al. 2011).

Total phenol content of the extract was 40.66 ± 2.5 μg GAE/250 μg extract, while total flavonoids content of the extract was 100.9 ± 8.7 μg CE/500 μg extract, whereas GAE is Gallic acid equivalent, and CE is Catechin equivalent.

### Phenolic compounds high-performance liquid chromatography (HPLC) analysis

#### Reagents and chemicals

All the HPLC solvents were purchased from Merck (Darmstadt, Germany). The standards of rosmarinic acid (RA), salvianolic acid B (SAL B), salvianolic acid A (SAL A) and caffeic acid (CA) were purchased from Sigma-Aldrich (Chicago, IL).

#### Preparation of standard solutions

The standard stock solutions were separately prepared in pure ethanol and diluted to appropriate concentration range for the establishment of calibration curves.

#### Phenolic compounds extraction from sample

The dried and powdered aerial parts of Lavender (0.5 g) were macerated with 10 ml methanol (for 24 h at room temperature in a dark place). After filtration, the solvent was evaporated in vacuum at 40 °C to obtain crude methanol extract. Dried extract of the sample was stored in the dark at 4 °C until tested. Ethanol extract solution of the sample was freshly prepared in 99% EtOH at 4 mg/mL concentration prior to measurement.

#### HPLC analysis

The content of phenolic compounds was measured by HPLC method. The HPLC apparatus was a Smartline model (Kenuer, Baden, Germany) with a quaternary pump and a reversed phase column C18 Eurospher-100 (5 μm particle, 125 mm × 4 mm) coupled with a UV–VIS detector (D-14163 model). Data were processed by Software ChromGate (V 3.1, Kenuer, Baden, Germany). Separation was performed using a mobile phase (concentration gradient). The mobile phase consisted of water with 0.2% glacial acetic acid (solvent A) and acetonitrile (solvent B). The flow rate was kept at 1 mL/min. Initial condition was A–B (90:10, v/v), and linearly changed to A–B (75:25, v/v) in 15 min. The percentage of mobile phase A decreased to 20% at 40 min and reached to 0% in 45 min. This ratio remained stable until 50 min and in the next 5 min, the percentage of mobile phase A increased linearly to 90%. The injection volume was 20 μL, and peaks were monitored at 280 nm (common UV absorbance maximum of the compounds). The sample was filtered through a hydrophilic PTFE membrane filter with a 0.45-μm pore size before injection. Peaks were identified by congruent retention times compared with those of standards. The content of each phenolic compound was calculated from the corresponding calibration curve. Evaluation of sample was repeated three times.

Phenolic compounds content of the extract (mg/g DW) were as follows: rosmarinic acid (RA): 2.063, caffeic acid (CA): 0.41, salvianolic acids A and B (SAL A & B): Not detected.

### Statistical analysis

The animal’s behavioural activities in Y maze, EPM tasks and FST were statistically analyzed with one-way analysis of variance (ANOVA). All results are expressed as mean ± SEM. According to the degrees of freedom (*F*), values for which *p* < 0.05 were regarded as statistically significant. Significant differences between individual groups were determined by Holm–Sidak *post hoc* test.

## Results

### Effect of different doses of lavender extract on spatial memory impairment induced by scopolamine in Y-maze task

**Figure 1.**
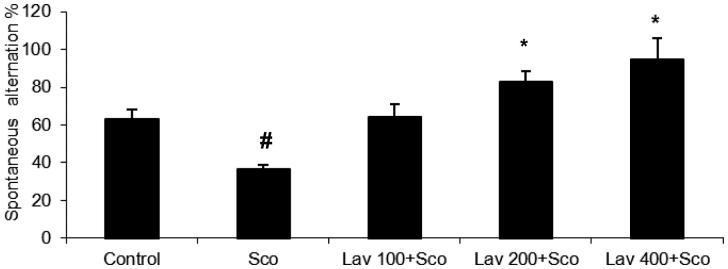
Effects of the scopolamine alone treatment and *Lavandula officinalis* extract pretreatment in different doses, on spontaneous alternation percentage in Y maze. Values are means ± SEM (*n* = 10 animals per group), #*p* ≤ 0.001 in compared with the control group. For post hoc analysis: Lav 200 + Sco and Lav400 + Sco versus Sco: *p ≤ 0.05. Sco: scopolamine; Lav: *L. officinalis*. Effects of the scopolamine alone treatment and *L. officinalis* extract pretreatment in different doses, on spontaneous alternation percentage in Y maze.

Figure 1 shows that 0.1 mg/kg scopolamine decreased significantly spatial memory in compared with the control group. Spatial memory in Y maze has been indicated by spontaneous alternation percentage. Kruskal–Wallis analysis shows that there are significant differences between groups *p* ≤ 0.001. Also *post hoc* Dunn’s method revealed significant statistical differences (*p* ≤ 0.05) between lavender pretreatment (200 and 400 mg/kg) scopolamine groups and scopolamine alone group. Results suggest that lavender extract in a dose-dependent manner have been improved scopolamine-induced memory impairment.

### Effect of different doses of lavender extract on anxiety in EPM task

**Figure 2.**
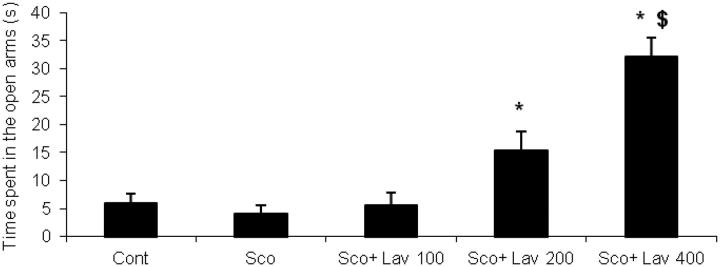
Effects of the scopolamine alone treatment and *Lavandula officinalis* extract pretreatment in different doses, on time spent on the open arms in elevated plus maze task. Values are means ± SEM (*n* = 10 animals per group), *p ≤ 0.001 in compared with control and scopolamine groups. For post hoc analysis: Lav 200 + Sco versus Lav400 + Sco: $*p* ≤ 0.05. Sco: scopolamine; Lav: *L. officinalis*. Effects of the scopolamine alone treatment and *L. officinalis* extract pretreatment in different doses, on time spent on the open arms in elevated plus maze task.

Behaviour in the EPM is mainly used to assess exploration and anxiety status. Figure 2 shows that 0.1 mg/kg scopolamine decreased non-significantly time spent on the open arms when compared with the control group. One-way ANOVA revealed that lavender extract pretreatment in 200 and 400 mg/kg enhanced significantly the time spent on the open arms when compared with control and scopolamine alone groups (*F* (4,45) = 20.47, *p* ≤ 0.001). Results suggest that lavender extract in a dose-dependent manner significantly diminished anxiety-like behaviour. Also, *post hoc* analysis revealed significantly statistical differences between 200 and 400 mg/kg pretreated groups (*p* ≤ 0.05).

### Effect of different doses of lavender extract ondepression-like behaviour in FST

**Figure 3.**
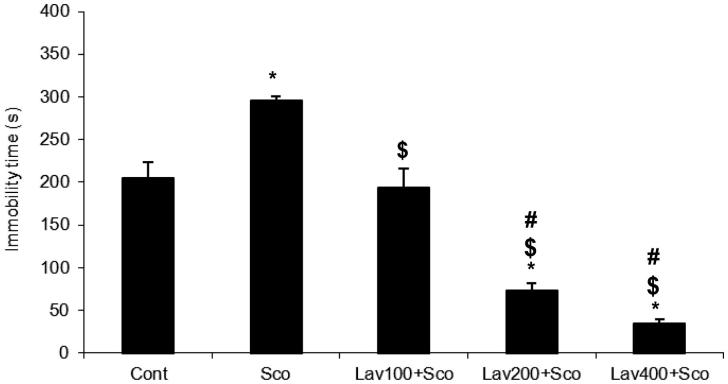
Effects of the scopolamine alone treatment and *Lavandula officinalis* extract pretreatment in different doses, on immobility time in forced swimming test. Values are means ± SEM (*n* = 10 animals per group), *p ≤ 0.001 in compared with the control group, $*p* ≤ 0.05 in compared with the scopolamine group and #*p* ≤ 0.05 in compared with 100 mg/kg lavender extract. Sco: scopolamine; Lav: *L. officinalis*. Effects of the scopolamine alone treatment and *L. officinalis* extract pretreatment in different doses, on immobility time in forced swimming test.

The possible antidepressant effects of the lavender extract were assessed by FST (Figure 3) and one-way ANOVA shows that in point of immobility time, there are statistically significant differences between groups (*F*(4,43) = 60.22, *p* ≤ 0.001). While immobility time has been enhanced by scopolamine, lavender extract with doses of 200 and 400 mg/kg reduced it significantly when compared with control and scopolamine alone groups. Also *post hoc* Holm–Sidak test revealed that there are significant differences between different doses of extract (200 and 400 mg/kg versus 100 mg/kg) (*p* ≤ 0.05). Results suggest that repeated application of scopolamine caused depression-like behaviour, and lavender extract improved it in a dose-dependent manner.

## Discussion

Anxiety and depression are common in AD patients, and are related to duration and severity of dementia (Garca-Alberca et al. 2011). In the present study, repeated application of 0.1 mg/kg scopolamine resulted in a significant decrease of spatial memory in Y maze (while there were no side effects on motor activity) and depressive-like behaviour in FST.

In accordance with our finding, many studies report memory impairment induced by scopolamine in Y maze (Hritcu et al. 2012; Baral et al. 2015; Foyet et al. 2015). Spatial memory in Y maze has been indicated by the percentage of spontaneous alternation. The Y-maze task is a specific and sensitive test of spatial recognition memory in rodents. The test relies on an innate tendency of rats to explore a novel environment. The Y maze used in this study involves no aversive stimuli and was considered suitable for evaluating memory (Hritcu et al. 2012).

Our findings also show that lavender extract improved spatial memory impairment induced by scopolamine, in a dose-dependent manner. These results are not in agreement with the previous study results. Hritcu et al. (2012) showed that inhalation of dried flower heads lavender essential oil vapours for a controlled time period (60 min) prior to performing behavioural testing, daily, for 7 continuous days did not display significantly any improvement effect on acquisition of the short-term memory of the scopolamine-treated rats (0.7 mg/kg) within Y-maze task. Difference in results may be related to the dose of scopolamine, the method of administration and the kind of the lavender extract.

Also, the present study demonstrates that scopolamine caused by anxiety and depression-like behaviour. In spite of some reports about rapid antidepressant efficacy of the scopolamine (Furey & Drevets 2006; Drevets & Furey 2010), our finding shows that repeated administration of scopolamine in rats (0.1 mg/kg) caused depression-like behaviour in FST.

The cholinergic system is one of the neurotransmitter systems implicated in the pathophysiological mechanism of mood disorders. Increasing cholinergic activity using physostigmine (an anticholinesterase inhibitor) provides a challenge uniquely capable of exacerbating depressive symptoms in depressed patients with major depressive disorder (MDD) and inducing depressive symptoms in manic patients with bipolar depression (BD). Also, it has been reported that intravenous injection of 4.0 μg/kg scopolamine in depressed patients showed antidepressant effects (Furey & Drevets 2006; Drevets & Furey 2010). Putative animal models of depression also have implicated the muscarinic system. The Porsolt ‘behavioural despair’ model of depression that uses the forced swim test is used broadly to evaluate the effect of pharmacologic agents on depressive behaviours. In the context of this model, antimuscarinic agents produced antidepressant-like effects. Moreover, rats bred selectively for increased sensitivity of muscarinic receptors showed putative behavioural analogues of depression, such as lethargy, reductions in self-stimulation and increased behavioural despair, in the forced swim test in response to cholinomimetic drugs (Furey & Drevets 2006; Voleti et al. 2013). Also, on one hand, it is demonstrated that scopolamine rapidly increases mammalian target of rapamycin complex 1 (mTORC1) signalling, synaptogenesis and antidepressant behavioural responses (Voleti et al. 2013).

On the other hand, it has been reported that intraperitoneal (i.p.) injection of single and repeated administration of scopolamine (0.7 mg/kg) caused anxiety and depressant-like behaviour in rats (Hritcu et al. 2012; Aydin et al. 2016). These results are agreement to our findings. Therefore, it seems that scopolamine administration in very low dose (4.0 or 25 μg/kg) exhibits anti depressive efficacy, while higher doses of scopolamine that induced memory impairment, shows anxiety and depressive-like behaviour in which must be investigated further.

The EPM is considered to be an etiologically valid animal model of anxiety because it uses natural stimuli (Grundmann et al. 2007). An anxiolytic agent increases the time spent on the open arms and the frequency of entries into the open arms in this test (Hritcu et al. 2012). In the present study, lavender extract pretreatment in 200 and 400 mg/kg in scopolamine-treated rats enhanced significantly the time spent on the open arms in comparison with the control and scopolamine alone groups. These results could be explained by an anxiolytic-like effect of the lavender extract in treated animals in a dose-dependent manner. So far, only one study has been used the EPM to test the effects of lavender oil inhalation in scopolamine-treated rats (Hritcu et al. 2012). Our results support anxiolytic effect of lavender in scopolamine-treated rats, in the previous study. Lavender essential oil has been shown to reduce anxiety in both clinical and preclinical studies. Preclinical studies have found anxiolytic effects of lavender essential oil in the Geller conflict test, Vogel test, EPM and open-field test (Chioca et al. 2013). Also, there is some evidence supporting the effectiveness of lavender oil in the treatment of anxiety in humans. Lehrner et al. (2005) reported that lavender odour was effective in relieving anxious mood in dental patients exposed to lavender in the waiting room. While in humans, cognitive mechanisms of odour transduction may complicate the study of pharmacological effects, animal studies give some evidences for herbal medicine extract having pharmacological properties. On the contrary, in this context, certain negative results have been reported. It has been showed that inhalation of lavender oil did not increase open arm explorations of mice tested on the EPM (Komiya et al. 2006). Hawken et al. (2012) observed that lavender essential oil exerted opposite effects in sheep for their stress response: lavender increases agitation, vocalization and escape attempts in isolated stressed ‘nervous sheep’ (sheep that exhibit increase motor activity and vocalization frequency when isolated or in human presence).

However, our findings could be explained by an anxiolytic-like effect of the lavender aerial part hydro-alcoholic extract in scopolamine-treated rats, in a dose-dependent manner. The lavender species most applied in anxiety-related studies is *L. angustifolia*, which contains linalool and linalyl acetate as its main components. Linalool has been the compound most linked to the anxiolytic effect of lavender. Moreover, it has been shown that the anxiolytic-like effect of linalool in mice tested in the EPM was potentiated by linalyl acetate (Chioca et al. 2013). However, in the present study, the concentration of linalool and linalyl acetate in *L. officinalis* aerial part hydroalcoholic extract was low or trace, while the extract showed anxiolytic-like effect. Therefore, it seems that other components may be also involved.

The FST has been validated as a suitable method for assessing the antidepressant properties of drugs. When rodents are forced to swim in a confined space, after an initial period of struggling, they would become immobile, resembling a state of despair and mental depression. This inescapable stressful situation can be evaluated by assessing different behavioural strategies (Hritcu et al. 2012). In the present study, while immobility time has been enhanced by scopolamine, lavender extract with doses of 200 and 400 mg/kg reduced it significantly compared with the control and scopolamine groups. Therefore, our finding suggests that repeated application of scopolamine caused depressant-like behaviour, and lavender extract improved it in a dose-dependent manner.

It is reported that lavender extract did not change the locomotor activity in the open-field test (Kageyamal et al. 2012). Therefore, on one hand, reduction of immobility time in FST by lavender extract explains antidepressant-like effects that are independent of motor stimulation. On the other hand, enhancement of immobility time by scopolamine was not a sedative behaviour because while 0.7 mg/kg scopolamine induced sedation (pilot study), 0.1 mg/kg scopolamine which has been used in these study, did not, and all experiments especially Y-maze task tested correctly.

Hritcu et al. (2012) showed that inhalation of dried flower heads lavender essential oil vapours in the scopolamine-treated rats, for controlled time period (60 min) prior to performing behavioural testing, daily, for 7 continuous days, was significantly reduced the immobility time in FST. Therefore, antidepressant-like effects of lavender aerial parts extract in our study are in agreement with these results in Hritcu et al. (2012) study. Although ustukhuddus have been used in depressed patients in Iranian traditional medicine (Ebn-e 1991), there are a few scientific documents on this subject. In this context, there are some studies about positive effects of lavender in decreasing the depression level in depressed patients (Nikfarjam et al. 2010, 2013). It has been shown that *L. angustifilia* infusion exhibited some positive therapeutic effects on depressed patients, by most importantly reduction of mean depression score, and might be used alone or as an adjunct to other anti-depressant drugs (Akhondzadeh et al. 2003; Nikfarjam et al. 2010). Thus, our findings support documents about anti-depressant properties of lavender.

The effects of lavender extract on anxiety and depression-like behaviours can be explained considering its multiple chemical constituents and their effects. Phytochemical analysis of the lavender extract in the present study revealed that aerial parts of the extract contained polyphenols, flavonoids, sterols, saponins and cardiac glycosides.

Some studies showed that phytochemicals like alkaloids, flavonoids, phenolic acids, lignans, cinnamates, terpenes and saponins possess anxiolytic effects in a wide range of animal models of anxiety (Farzaei et al. 2016). The inhibitory effects of flavonoids on monoamine oxidases (MAOs) have attracted great interest, since alterations in monoaminergic transmission are reported to be related to neurodegenerative diseases such as Parkinson's and ADs and psychiatric disorders such as depression and anxiety, thus MAOs may be considered as targets for the treatment of these multi-factorial diseases (Turkmenoglu et al. 2015). The natural flavonoids compound luteolin (2-(3,4-Dihydroxyphenyl)-5,7-dihydroxy-4-chromenone) has been reported to have antidepressant, antinociceptive and anxiolytic-like effects, which possibly involve the mechanisms of modulating GABA signalling (Shen et al. 2016). Polyphenolic compounds are phytochemicals known for their biological antioxidative, neuroprotective and cognitive properties. For instance, it has been shown that different polyphenols can increase synaptic plasticity in the context of adult hippocampal neurogenesis (AHN) and also promote hippocampal long-term potentiation. In addition, it has been verified that polyphenols can enhance learning and memory and reduce the risk of developing age-related neurodegenerative diseases, possibly via a decrease in reactive oxygen species (ROS) production and inflammation in models of aging. Besides antidepressant drugs, different polyphenolic compounds such as catechins, curcumin and resveratrol have been observed to have antidepresssant-like effects in rodents and human. This suggests that polyphenols could be key compounds for the improvement of psychiatric disorders like depression or anxiety. The facts that polyphenols have been shown to be helpful compounds against depression and that they can increase AHN suggest that these molecules might affect mood, and not only cognition, via AHN (Dias et al. 2012).

Oxidative stress has been associated with the pathogenesis of several diseases including psychiatric disorders and has recently been implicated in depression and anxiety. Depression and anxiety are related to lowered plasma concentrations of antioxidants, such as vitamin E, tryptophan, tyrosine, albumin, zinc, glutathione and lowered antioxidative enzyme activities (Grases et al. 2014)**.** Oxidative stress and perturbation of redox balance can have significant consequences in AHN and possibly, in hippocampal-dependent functions (Huang et al. 2012).

AHN is a widely observed phenomenon verified in different adult mammalian species including humans. Factors such as environmental enrichment, voluntary exercise and diet have been linked to increased levels of AHN. Conversely, aging, stress, anxiety and depression have been suggested to hinder it. However, the mechanisms underlying these effects are still unclear and yet to be determined. It is suggested that diet polyphenols with antioxidant properties exert positive effects on anxiety and depression, possibly in part via regulation of AHN (Dias et al. 2012). Among the phenolic compounds rosmarinic acid and caffeic acid, a major metabolite of rosmarinic acid have been reported to have an antidepressive and anxiolytic-like effects (Takeda et al. 2002, 2003; Ibarra et al. 2010; Kageyamal et al. 2012). Therefore, in the present study, rosmarinic acid and caffeic acid seem to be two of the active compounds for the anxiolytic and antidepressant-like effects of lavender extract.

## References

[CIT0001] Abioye EO, Akinpelu DA, Aiyegoro OA, Adegboye MF, Oni MO, Okoh AI. 2013 Preliminary phytochemical screening and antibacterial properties of crudestem bark extracts and fractions of *Parkia biglobosa* (Jacq.). Molecules. 18:8485–8499. 23873387 10.3390/molecules18078485 PMC6269791

[CIT0002] Akhondzadeh SH, Kashani L, Fotouhi A, Jarvandi S, Mobaseri M, Moin M, Khani M, Jamshidi AH, Baghalian K, Taghizadeh M. 2003 Comparison of *Lavandula angustifolia* Mill. Tincture and imipramine in the treatment of mild to moderate depression: a double-blind, randomized trial. Prog Neuropsychopharmacol Biol Psychiatry. 27:123–127. 12551734 10.1016/s0278-5846(02)00342-1

[CIT0003] Aydin E, Hritcu L, Dogan G, Hayta S, Bagci E. 2016 The effects of inhaled *Pimpinella peregrina* essential oil on scopolamine-induced memory impairment, anxiety, and depression in laboratory rats. Mol Neurobiol. 2016:1–11. 10.1007/s12035-016-9693-9 26768430

[CIT0004] Baral S, Cho DH, Pariyar R, Yoon CS, Chang BY, Kim DS, Cho HK, Kim SY, Oh H, Kim YC, et al 2015 The ameliorating effect of myrrh on scopolamine-induced memory impairments in mice. Evid Based Complement Alternat Med. 2015:1–9. 10.1155/2015/925432 PMC4655272 26635888

[CIT0005] Bradley BF, Starkey NJ, Brown SL, Lea RW. 2007 Anxiolytic effects of *Lavandula angustifolia* odour on the Mongolian gerbil elevated plus maze. J Ethnopharmacol. 111:517–525. 17289317 10.1016/j.jep.2006.12.021

[CIT0006] Burda K, Czubak A, Kus K, Nowakowska E, Ratajczak P, Zin J. 2011 Influence of aripiprazole on the antidepressant, anxiolytic and cognitive functions of rats. Pharmacol Rep. 63:898–907. 22001977 10.1016/s1734-1140(11)70605-3

[CIT0007] Chioca LR, Ferro MM, Baretta IP, Oliveira SM, Silva CR, Ferreira J, Losso EM, Andreatini R. 2013 Anxiolytic-like effect of lavender essential oil inhalation in mice: participation of serotonergic but not GABAA/benzodiazepine neurotransmission. J Ethnopharmacol. 147:412–418. 23524167 10.1016/j.jep.2013.03.028

[CIT0008] Coplan JD, Aaronson CJ, Panthangi V, Kim Y. 2015 Treating comorbid anxiety and depression: Psychosocial and pharmacological approaches. World J Psychiatry. 5:366–378. 26740928 10.5498/wjp.v5.i4.366 PMC4694550

[CIT0009] Dias GP, Cavegn N, Nix A, do Nascimento Bevilaqua MC, Stangl D, Zainuddin MS, Nardi AE, Gardino PF, Thuret S. 2012 The role of dietary polyphenols on adult hippocampal neurogenesis: molecular mechanisms and behavioural effects on depression and anxiety. Oxid Med Cell Longev. 2012:1–18. 10.1155/2012/541971 PMC3395274 22829957

[CIT0010] Drevets WC, Furey ML. 2010 Replication of scopolamine's antidepressant efficacy in major depressive disorder: a randomized, placebo-controlled clinical trial. Biol Psychiatry. 67:432–438. 20074703 10.1016/j.biopsych.2009.11.021 PMC3264395

[CIT0011] Dwyer AV, Whitten DL, Hawrelak JA. 2011 Herbal medicines, other than St. John's Wort, in the treatment of depression: a systematic review. Altern Med Rev. 16:40–49. 21438645

[CIT0012] Ebn-e SA. 1991 Ghanoon dar Teb. Tehran, Iran: Soroosh Press (In Persian).

[CIT0013] Effati-Daryani F, Mohammad-Alizadeh S, Mirghafourvand M, Taghizadeh M, Mohammadi A. 2015 Effect of lavender cream with or without foot-bath on anxiety, stress and depression in pregnancy: a randomized placebo-controlled trial. J Caring Sci. 4:63–73. 25821760 10.5681/jcs.2015.007 PMC4363653

[CIT0014] Farzaei MH, Bahramsoltani R, Rahimi R, Abbasabadi F, Abdollahi M. 2016 A systematic review of plant-derived natural compounds for anxiety disorders. Curr Top Med Chem. 16:1924–1942. 26845556 10.2174/1568026616666160204121039

[CIT0015] Fernandes JS, Mori MA, Ekuni R, Oliveira RM, Milani H. 2008 Long-term treatment with fish oil prevents memory impairments but not hippocampal damage in rats subjected to transient, global cerebral ischemia. Nutr Res. 28:798–808. 19083490 10.1016/j.nutres.2008.09.004

[CIT0016] Fißler M, Quante A. 2014 A case series on the use of lavendula oil capsules in patients suffering from major depressive disorder and symptoms of psychomotor agitation, insomnia and anxiety. Complement Ther Med. 22:63–69. 24559818 10.1016/j.ctim.2013.11.008

[CIT0017] Foyet HS, Ngatanko Abaïssou HH, Wado E, Acha EA, Alin C. 2015 *Emilia coccinae* (SIMS) G extract improves memory impairment, cholinergic dysfunction, and oxidative stress damage in scopolamine-treated rats. BMC Complement Altern Med. 23:1–12. 10.1186/s12906-015-0864-4 PMC4580266 26400617

[CIT0018] Furey ML, Drevets WC. 2006 Antidepressant efficacy of the antimuscarinic drug scopolamine: a randomized, placebo-controlled clinical trial. Arch Gen Psychiatry. 63:1121–1129. 17015814 10.1001/archpsyc.63.10.1121 PMC3250308

[CIT0019] Garca-Alberca JM, Lara JP, Berthier ML. 2011 Anxiety and depression in caregivers are associated with patient and caregiver characteristics in Alzheimer's disease. Int J Psychiatry Med. 41:57–69. 21495522 10.2190/PM.41.1.f

[CIT0020] Gilani AH, Rahman A. 2005 Trends in ethnopharmocology. J Ethnopharmacol. 100:43–49. 16127805 10.1016/j.jep.2005.06.001

[CIT0021] Grases G, Colom MA, Fernandez RA, Costa-Bauzá A, Grases F. 2014 Evidence of higher oxidative status in depression and anxiety. Oxid Med Cell Longev. 2014:1–5. 10.1155/2014/430216 PMC4020168 24876911

[CIT0022] Grundmann O, Nakajima J, Seo S, Butterweck V. 2007 Anti-anxiety effects of *Apocynum venetum* L. in the elevated plus maze test. J Ethnopharmacol. 110:406–411. 17101250 10.1016/j.jep.2006.09.035

[CIT0023] Hawken PA, Fiol C, Blache D. 2012 Genetic differences in temperament determine whether lavender oil alleviates or exacerbates anxiety in sheep. Physiol Behav. 105:1117–1123. 22192707 10.1016/j.physbeh.2011.12.005

[CIT0024] Hritcu L, Cioanca O, Hancianu M. 2012 Effects of lavender oil inhalation on improving scopolamine-induced spatial memory impairment in laboratory rats. Phytomedicine. 19:529–534. 22402245 10.1016/j.phymed.2012.02.002

[CIT0025] Huang TT, Zou Y, Corniola R. 2012 Oxidative stress and adult neurogenesis-effects of radiation and superoxide dismutase deficiency. Semin Cell Dev Biol. 23:738–744. 22521481 10.1016/j.semcdb.2012.04.003 PMC3410958

[CIT0026] Ibarra A, Feuillere N, Roller M, Lesburgere E, Beracochea D. 2010 Effects of chronic administration of *Melissa officinalis* L. extract on anxiety-like reactivity and on circadian and exploratory activities in mice. Phytomedicine. 17:397–403. 20171069 10.1016/j.phymed.2010.01.012

[CIT0027] Jia Z, Tang M, Wu J. 1999 The determination of flavonoid contents in mulberry and their scavenging effects on superoxide radicals. Food Chem. 64:555–559.

[CIT0028] Kageyamal A, Ueno T, Oshio M, Masuda H, Horiuchi H, Yokogoshi H. 2012 Antidepressant-like effects of an aqueous extract of lavender (*Lavandula angustifolia* Mill.) in rats. Food Sci Technol Res. 18:473–479.

[CIT0029] Kashani MS, Tavirani MR, Talaei SA, Salami M. 2011 Aqueous extract of lavender (*Lavandula angustifolia*) improves the spatial performance of a rat model of Alzheimer's disease. Neurosci Bull. 27:99–106. 21441971 10.1007/s12264-011-1149-7 PMC5560344

[CIT0030] Kasper S. 2015 Phytopharmaceutical treatment of anxiety, depression, and dementia in the elderly: evidence from randomized, controlled clinical trials. Wien Med Wochenschr. 165:217–228. 26092515 10.1007/s10354-015-0360-y

[CIT0031] Kay C, Haper DN, Hunt M. 2010 Differential effects of MDMA and scopolamine on working versus reference memory in the radial arm maze task. Neurobiol Learn Mem. 93:151–156. 19766200 10.1016/j.nlm.2009.09.005

[CIT0032] Klinkenberg I, Blokland A. 2010 The validity of scopolamine as a pharmacological model for cognitive impairment: a review of animal behavioral studies. Neurosci Biobehav Rev. 34:1307–1350. 20398692 10.1016/j.neubiorev.2010.04.001

[CIT0033] Komiya M, Takeuchi T, Harada E. 2006 Lemon oil vapor causes an anti-stress effect via modulating the 5-HT and DA activities in mice. Behav Brain Res. 172:240–249. 16780969 10.1016/j.bbr.2006.05.006

[CIT0034] LaGow B, Gruenwald J, Brendler T, Jaenicke C. 2004. PDR for Herbal Medicines, USA. Thomson Herbal Monographs, Cornell University.

[CIT0035] Lehrner J, Marwinski G, Lehr S, Johren P, Deecke L. 2005 Ambient odors of orange and lavender reduce anxiety and improve mood in a dental office. Physiol Behav. 86:92–95. 16095639 10.1016/j.physbeh.2005.06.031

[CIT0036] Müller LG, Salles LA, Stein AC, Betti AH, Sakamoto S, Cassel E, Vargas RF, Von Poser GL, Rates SM. 2012 Antidepressant-like effect of *Valeriana glechomifolia* Meyer (Valerianaceae) in mice. Prog Neuropsychopharmacol Biol Psychiatry. 36:101–109. 21889562 10.1016/j.pnpbp.2011.08.015

[CIT0037] Nikfarjam M, Parvin N, Asarzadegan N. 2010 The effect of *Lavandula angustifolia* in the treatment of mild to moderate depression. J Shahrekord Univ Med Sci. 11:66–73.

[CIT0038] Nikfarjam M, Parvin N, Assarzadegan N, Asghari S. 2013 The effects of *Lavandula angustifolia* Mill infusion on depression in patients using citalopram: a comparison study. Iran Red Crescent Med J. 15:734–739. 24578844 10.5812/ircmj.4173 PMC3918201

[CIT0039] Perry R, Terry R, Watson LK, Ernst E. 2012 Is lavender an anxiolytic drug? A systematic review of randomised clinical trials. Phytomedicine. 19:825–835. 22464012 10.1016/j.phymed.2012.02.013

[CIT0040] Popović M, Giménez de Béjar V, Popović N, Caballero-Bleda M. 2015 Time course of scopolamine effect on memory consolidation and forgetting in rats. Neurobiol Learn Mem. 118:49–54. 25460041 10.1016/j.nlm.2014.11.006

[CIT0041] Rahmati B, Khalili M, Roghani M, Ahghari P. 2012 Anticonvulsant effect of hydro-alcoholic extract of *Lavandula officinalis* on seizures in pentylenetetrazol-induced kindling model in male rat. Danesh Med 19:1–9. (In Persian).

[CIT0042] Rahmati B, Khalili M, Roghani M, Ahghari P. 2013 Anti-epileptogenic and antioxidant effect of *Lavandula officinalis* aerial part extract against pentylenetetrazol-induced kindling in male mice. J Ethnopharmacol. 148:152–157. 23603193 10.1016/j.jep.2013.04.004

[CIT0043] Rimjhim S, Kumari N, Jainendra K. 2014 Preliminary phytochemical screening of methanolic extract of *Clerodendron infortunatum* . IOSR J Applied Chem. 7:10–13.

[CIT0044] Shams Ardekani MR, Hajimahmoodi M, Oveisi MR, Sadeghi N, Jannat B, Ranjbar AM, Gholam N, Moridi T. 2011 Comparative antioxidant activity and total flavonoid content of Persian Pomegranate (*Punica granatum* L.) cultivars. Iranian J Pharma Res. 10:519–524. PMC3813023 24250384

[CIT0045] Sharifzadeh M, Hadjiakhoondi A, Khanavi M, Susanabadi M. 2006 Effects of aqueous, methanolic and chloroform extracts of rhizome and aerial parts of *Valeriana officinalis* L. on naloxone-induced jumping in morphine dependent mice. Addict Biol. 11:145–151. 16800827 10.1111/j.1369-1600.2006.00016.x

[CIT0046] Shen ML, Wang CH, Chen RY, Zhou N, Kao ST, Wu DC. 2016 Luteolin inhibits GABAA receptors in HEK cells and brain slices. Sci Rep. 6:27695. 27292079 10.1038/srep27695 PMC4904371

[CIT0047] Takeda H, Tsuji M, Inazu M, Egashira T, Matsumiya T. 2002 Rosmarinic acid and caffeic acid produce antidepressive-like effect in the forced swimming test in mice. Eur J Pharmacol. 449:261–267. 12167468 10.1016/s0014-2999(02)02037-x

[CIT0048] Takeda H, Tsuji M, Miyamoto J, Masuya J, Iimori M, Matsumiya T. 2003 Caffeic acid produces antidepressive- and/or anxiolytic-like effects through indirect modulation of the alpha 1A-adrenoceptor system in mice. Neuroreport. 14:1067–1070. 12802204 10.1097/01.wnr.0000073427.02536.b0

[CIT0049] Tegegne MT, Mossie TB, Awoke AA, Assaye AM, Gebrie BT, Eshetu DA. 2015 Depression and anxiety disorder among epileptic people at Amanuel Specialized Mental Hospital, Addis Ababa, Ethiopia. BMC Psychiat. 15:17. 10.1186/s12888-015-0589-4 PMC4556015 26328614

[CIT0050] Turkmenoglu FP, Baysal İ, Ciftci-Yabanoglu S, Yelekci K, Temel H, Paşa S, Ezer N, Çalış İ, Ucar G. 2015 Flavonoids from *Sideritis* Species: human monoamine oxidase (hMAO) inhibitory activities, molecular docking studies and crystal structure of xanthomicrol. Molecules. 20:7454–7473. 25915461 10.3390/molecules20057454 PMC6272178

[CIT0051] Velioglu YS, Mazza G, Gao L, Oomah BD. 1998 Antioxidant activity and total phenolics in selected fruits, vegetables and grain products. J Agricul Food Chem. 46:4113–4117.

[CIT0052] Voleti B, Navarria A, Liu RJ, Banasr M, Li N, Terwilliger R, Sanacora G, Eid T, Aghajanian G, Duman RS. 2013 Scopolamine rapidly increases mammalian target of rapamycin complex 1 signaling, synaptogenesis, and antidepressant behavioral responses. Biol Psychiatry. 74:742–749. 23751205 10.1016/j.biopsych.2013.04.025 PMC3773272

[CIT0053] Xiang GQ, Tang SS, Jiang LY, Hong H, Li Q, Wang C, Wang XY, Zhang TT, Yin L. 2012 PPARγ agonist pioglitazone improves scopolamine-induced memory impairment in mice. J Pharm Pharmacol. 64:589–596. 22420664 10.1111/j.2042-7158.2011.01432.x

